# Cancer and Its Modern Treatment

**Published:** 1907-01-26

**Authors:** John A. Shaw-Mackenzie


### Hospital Clinics.

### CANCER AND ITS MODERN TREATMENT.

By JOHN A. SHAW-MACKENZIE, M.D.(Lond.)

The avidity with which the public Press seizes upon anything which promises to be sensational with respect to cancer has again been proved during the past few weeks by the publication in an evening journal of some references to trypsin. It is, however, much to be regretted that the editors of lay journals, when dealing with medical matters which may be assumed to be of public interest, do not take their information from the professional organs. Were this to be done, it is quite certain that much misconception would cease to be caused, and the public would not be misled by statements which the profession knows to be premature. So much attention, however, has recently been drawn to the question of the trypsin treatment of cancer that, perhaps, a few remarks thereon, briefly incorporating my personal views, may not be without interest to the readers of The Hospital.

The fact being now generally conceded that the parasitic theory of cancer is dead, we must turn elsewhere to seek for the solution of the etiology of the disease. In this connection it is just possible that the chemico-biological theory will prove in the end to be the solution of the problem. This theory seeks to establish that cancer is really a disease of faulty nutrition: that the failure of nutrition depends upon the loss of function, chiefly, of a particular organ—the pancreas; that owing to this defect cell-growth proceeds uncontrolled in some tissue of selection, with the result that the features of heterogeneous formations known as malignant growths, become manifest.

My attention was first drawn to the subject of the etiology of cancer by the fact that I found diabetes and cancer alternately occurring among the members of different families. That is say, it is quite frequent to find a cancerous patient allege that one of more of his relatives has died of diabetes. Remembering, then, that the pancreas is generally admitted to be the organ at fault in glycosuria, it occurred to me that possibly the same organ might be concerned in the evolution of cancer. Acting upon this hypothesis I proceeded to ascertain the chemical properties of various ferments, among which were included the pancreatic ferments. In the course of many investigations undertaken for me by Mr. F. W. Gamble, it was found, among other aids, that trypsin dissolved glycogen. This seemed to be a discovery of much significance, inasmuch as original research had previously disclosed that an excess of glycogen was present in malignant growths, and that the same excess was apparent in all rapidly developing embryonic tissues. Here, then, was a problem the solution of which appeared to be comparatively simple. The presence of an excess of glycogen is necessary as a nutriment to rapidly growing cells; in other words, cell growth depends upon the presence of glycogen in the tissues—it is Nature's food, without which cell-growth is impossible. Inductively, therefore, it seemed that if some means could be devised for interrupting or destroying this food supply to the cells, out of which cancerous tumours are formed, the cells would cease to proliferate, become atrophied, and die. Hence it was that my attention was directed to trypsin—the proteolytic ferment of the pancreas, whose property, without doubt, of breaking up glycogen in living tissues seemed to furnish the means by which the arrest of the development of malignant neoplasms could be accomplished.

Having arrived at these conclusions, I naturally lost no time in submitting them to the test of clinical investigation. Accordingly, on January 19, 1905—two years ago—I began the trial, in cases of inoperable carcinoma, of hypodermic injections of trypsin, combined with the internal administration and local application of pancreatic preparations.

Previously, however, to carrying out this treatment, I may add that many details of technique required to be determined. For example, among the first which demanded attention were the difficulties of preparing a solution of trypsin suitable for hypodermic injection, and of deciding upon the dosage which should be employed. There was nothing to guide one in the matter. Nevertheless the time occupied in arriving at a decision thereupon was not by any means lost, inasmuch as further investigation revealed several important features in respect to the nature and chemical properties of trypsin. Curious as it may seem, the precise chemical composition of this ferment has so far defied analysis. Again, its proteolytic activity varies; time and exposure to air decreases its activity, while heat, excess of water, carbonate of sodium, and various other combinations destroy it. A 5-per-cent. solution keeps better than a solution of 2 per cent.; the latter is soon apt to become cloudy. Bearing, then, in mind these various facts, obviously it is of the utmost importance that the solution of trypsin employed should be one the potency of which can be relied upon, and one in which confidence can be placed in the mode of its preparation. It is for this reason that I always use the preparations of trypsin supplied by Messrs. Allen and Hanburys, in whose laboratories, under the direction of Mr. Gamble, all the inquiries respecting the natural properties of trypsin were carried out. I ultimately decided that a freshly prepared 2-per-cent. solution was the best to commence with, and that 15 minims of this should be injected once daily.

Latterly, in addition, I have been using, in 2-4 drachm doses, preparations of intestinal glands, containing, as I understand, secretin, or "erepsin," and "enterokinose" (Allen and Hanburys) alternately with trypsin.

It should be clearly understood that the so-called trypsin treatment of cancer does not merely include the administration of trypsin by the mouth or hypodermically. For example, a very useful, if not indispensable, adjunct to the treatment, is that of the hypodermic administration of chian turpentine, as introduced in 1904 by Colonel T. Ligertwood, C.B., M.D., and myself. Cases of carcinoma in which the blood-count has been taken during the administration of trypsin show that marked increased leucocytosis ensues if at the same time chian turpentine be thus used. To strengthen, then, and increase the phagocytic action of the white blood-cells must materially assist the potency of the trypsin. In my opinion, however, in every case of cancer which comes under treatment, a blood-examination should be regularly carried out; the importance of this is just as great as that of regularly examining the urine in cases of albuminuria.

I desire to direct attention to the value of purified ox-gall which I learned from the work of Mr. J. H. Webb (Melbourne). He gives it by the mouth in conjunction with subcutaneous injections of sodium oleate (soap) solution, and there are few cases in which I do not use the ox-gall or alternate treatment. He claims for his method apparent arrest of growth, relief of pain and removal of fœtor, and has recorded some remarkable results, notably in recurrent carcinoma of the breast and in superficial epitheliomata. Mr. Webb's theory is that in carcinoma the liver is at fault with some defect in the biliary secretion permitting cholesterin to separate, and that bile salts and soap are a solvent of cholesterin.

Again, I lay much stress upon a special quantity of sugar being consumed during the course of the treatment. It is at least possible, not to say probable, that the fœtal glycogen, as also of that in diabetes and carcinoma, arises in part from a splitting-up of proteids, while probably this result may be reduced by the action of carbohydrate food and sugar which decrease the waste of cell-proteids, as pointed out by Herter. In this connection an interesting fact was revealed by a patient who has done well under the trypsin treatment. He was referred to me with inoperable malignant disease, and it was found on examination that besides his carcinoma he was suffering from glycosuria. Despite this complication, however, I ordered the usual quantity of sugar daily; in the course of a few days his urine was found to be absolutely free from sugar. This most significant fact raises the question of the possible good results which might accrue to diabetic patients by treating them with trypsin or erepsin and, as the above case would seem to suggest, allowing them at the same time to indulge in a saccharine diet. What a boon this would be in the treatment of diabetes none but a diabetic patient could possibly estimate. The monotony, insipidity, and ultimate distastefulness of a purely diabetic diet is a feature of the disease which presses very hardly upon the hapless sufferers therefrom. Although I have written and published much regarding the possibilities of trypsin in this connection, I cannot find that the treatment has ever been given a trial. This is somewhat surprising having regard to the irremediable nature of the disease, and the little that can be done for it by treatment. I cannot help thinking that it would be well to submit a series of diabetic patients to the trypsin treatment, in order to determine its value, especially in view of the close connection which appears to exist between diabetes and cancer.

I have now to offer a few remarks upon another aspect of this question—namely, in reference to the cases of carcinoma suitable for the trypsin treatment. In this regard, although it is a fact that the treatment has mainly been discussed in connection with so-called inoperable cases of cancer, it is nevertheless true that the nutritional defect, to which I attribute the origin of the disease, is present in all the cases, and therefore in every case the treatment should be commenced directly the presence of carcinoma has been determined. If the patient has undergone surgical treatment, then the trypsin should be administered as soon as possible after the operation. Time is undoubtedly valuable in these cases—the earlier in the course of the disease that the trypsin method is resorted to, the better, in my experience, is the result. On the other hand, it is impossible to expect any improvement in the last sad stages of the disease. Repeatedlv within the past two years I have been called upon to advise treatment in such circumstances. But trypsin, like anything else would be, is powerless to effect good after auto-intoxication from an ulcerating septic, cancerous growth has supervened, or again, after multiple manifestations of the disease have involved the organs of the body generally.

And yet, as I have seen, under the most unfavourable conditions some relief has been obtained from trypsin. I am now more particularly referring to those cases in which a foul cancerous wound is present, accompanied by an overpowering fœtor, which not only harasses the patient by the intensity of its repulsiveness, but renders the attentions of nurses and others a matter of much difficulty and self-denial for the same reason. My experience of trypsin is that it arrests all this fœtor. If the trypsin treatment can do this, even if it cannot do more for the patient, I doubt not that concurrent opinion will be in favour of regarding its introduction as, at all events, of some benefit in the treatment of advanced cancerous disease.

But as I have observed above, it is impossible to expect any material improvement in the advanced cases, and it is, comparatively speaking, uncommon for the inoperable cases to be sufficiently uncomplicated to afford the trypsin treatment a reasonable prospect of yielding a good result. Still I can only add that it has been among many of the inoperable cases that the most gratifying results have been obtained. Those of my cases which have been recorded will be found fully described in my book "The Nature and Treatment of Cancer," and I am now preparing for publication a further series in which the good effects of the treatment are, perhaps, more manifestly displayed than are shown in the cases which have already been published. No doubt, as further experience of this treatment is gained, we shall later on be in a better position to judge of the class of cases in which good results may be anticipated. So far as I have been able to arrive at a conclusion in the matter, I would say that those patients are the most favourable for treatment in whom the disease has been of short duration and is limited in extent.

Under favourable conditions the good effects of the treatment are illustrated as follows: (1) Alleviation of pain and pressure symptoms; (2) arrest of fœtor in the case of ulcerated wounds; (3) shrinkage and apparent arrest of the growth; (4) changes in the growth pointing to diminution of infiltration and induration; (5) marked improvement in the general nutrition, as is evidenced by the return of the appetite and a progressive increase in the body weight. My experience has so far shown that in all save the most hopeless cases these features invariably result from the trypsin treatment. The recovery and increase of weight which ensue is perhaps the most remarkable of the good effects noticed. Presumably this may partly be due to the amount of sugar which is added to the diet, and yet it is essential to remember that the improved general nutrition could not have any influence in arresting the progress of the disease; on the contrary, we should expect the effect to be just the reverse.

Reverting to the subject of sugar, I have already noted in my work the curious fact that in cases of cancer it is quite common to elicit the history of a natural objection to sugar generally. In some cases it is avoided, in a precautionary sense, owing to the death of a member of the family from diabetes. In most cases in women it is eliminated from the diet as conducing to obesity; in other cases the medical practitioner prohibits it, in consequence of the tendency of the patients to gout or rheumatism. Whether there is or is not any real connection between the avoidance of sugar and the onset of cancer, it is impossible to avoid the reflection that the tendency of the age to cease taking sugar as much as possible, either in solid or liquid forms—the latter, for example, in the form of sweet wines—may have had something to do with the increase of cancer during recent years.

In mentioning these and other similar facts, and recalling how much of our so-called knowledge of cancer is based upon mere hearsay evidence, or even upon tradition, I am again reminded of the valuable suggestion of my friend, Mr. Percy Dunn, F.R.C.S., in an article in the *Nineteenth Century* for 1893, in favour of the appointment of a Royal Commission on Cancer. In view of the alarmingly progressive increase in its annual mortality, the lack of knowledge which prevails respecting the details of its incidence—so far as climate, locality, domestic environment, and other possible contributory causes are concerned—I entirely agree with Mr. Dunn that nothing short of a Royal Commission of Inquiry could possibly elicit the information required. In the House of Commons some years ago the statement was made that such a Commission was unnecessary. No doubt this statement was based upon the advice of some official to whom the then Prime Minister applied for guidance. I venture, however, emphatically to assert that Mr. Balfour was wrongly advised. It is undeniably true that had the appointment of a Commission then been conceded, we should probably now be in possession of many facts of which much use could be made, in a preventive sense; but as it is, everything which relates to the contributory causes underlying the incidence of cancer in this country is still in a state of chaos, still dependent upon tradition, still environed with the ambiguity which springs from undetermined facts, merely because no official or authorised investigation has been carried out to collect and sift the evidence bearing thereon. Malignant disease is rapidly becoming one of the most frequent causes of death in this country, and yet we are assured that no inquiry is needed to determine the possible causes of this increasing mortality. No one now believes that the increase, as shown by the Registrar-General's Returns, is due merely to the better registration of the fatal cases of cancer. Every middle-aged medical man in active practice in this Kingdom has almost daily confirmation of the fact that he sees more cases of carcinoma among his patients than was the case some years ago. And yet, despite all this, I again repeat we are informed that there is no necessity for any official inquiry. The last Government having failed to yield to the expressed desire for a Royal Commission on Cancer, strong efforts should be made with the present Government to grant so urgent a demand in the interests of the whole community. For, after all, let it be remembered, no matter how successful our treatment of the disease may be in the future, it is the policy of the prevention of cancer upon which our minds should be mainly concentrated.

It is usual in the present day to speak of the predisposing cause of carcinoma, among which are included conditions which are supposed to act as sources of irritation to the tissues of the body. In this connection one may recall the frequency with which epithelioma of the lower lip seems to be associated with the smoking of a clay pipe; or the tendency to epithelioma of the tongue which follows the irritation of the jagged edge of a tooth; or again, the development of mammary cancer which is seen after chronic eczema of the nipple. Cancer of the breast, also, is supposed by some to be connected with the pressure of the corsets; an assertion presumably based upon the fact that mammary carcinoma is practically unknown among native women who do not use such an appliance. My impression, however, is, that if pressure under these circumstances can be regarded as a predisposing cause of cancer, it is the nipple, not so much as the breast, which suffers in consequence. Moreover, how uncommon it is for women to devote ordinary hygienic attention to the nipple. Every woman should cleanse the nipples daily; quite possibly the neglect to do so is in time followed by the absorption of septic matter, which has the effect of keeping the breasts in an irritative condition without actually leading to the development of inflammatory signs.

Furthermore, I have witnessed that common affection of the enlargement of the axillary glands associated with "lumpy" breast, at the menstrual period, rapidly subside as the result of the application of antiseptic lotions to the nipple. In obstetric practice, of course, the nipple receives much attention during pregnancy and before actual labour commences. But apparently it is only at such times that this very necessary detail in the personal hygiene of women is properly and regularly observed.

In the course of the past few years I have been more and more impressed with the absence of facilities for the treatment of poor persons suffering from inoperable carcinoma. In illustration thereof I will give an instance which has come under my notice within the past few days. I was asked to see a poor woman suffering from inoperable cancer of the left breast involving the shoulder and the supraclavicular region. The history showed that she had had "lumps" in her breast for forty years, but had never paid much attention to them. Her youngest brother died at the age of 45 from diabetes—a significant fact. She herself had never been a sugar-eater; all her life sweet things had caused her to feel sick. But here was this poor patient, the victim of advanced carcinoma, with nothing but the inadequate and unsuitable surroundings of her own home to meet the necessities of her case. Her hopeless condition rendered her ineligible for the wards of a general hospital or for any other similar institution. There is a Home for the Dying, an admirable institution of its kind, but her condition was scarcely sufficiently desperate for this; nor could she be regarded as a pauper for whom the excellent Poor-law Infirmaries provide ample accommodation. So many cases of this description have of late come under my notice that I am strongly of the opinion that some special institution is urgently required for the treatment of these cases—apart from the general hospitals. Such a Home would provide poor patients with all the best and latest methods for the alleviation of their melancholy and unfortunate state.

